# Supplementary material for: Engineered gut symbionts mediate cross-phylum antagonism to suppress uropathogenic Escherichia coli colonization
Source: bioRxiv. 2026 May 12:2026.05.11.724322. Preprint. [Version 1] doi: 10.64898/2026.05.11.724322 (PMC13192842; doi:10.64898/2026.05.11.724322)
Supplement: 1 [file NIHPP2026.05.11.724322V1-supplement-1.pdf]

bioRxiv preprint doi: https://doi.org/10.64898/2026.05.11.724322; this version posted May 12, 2026. The copyright holder for this preprint (which was not certified by peer review) is the author/funder, who has granted bioRxiv a license to display the preprint in perpetuity. It is made available under a CC-BY 4.0 International license.

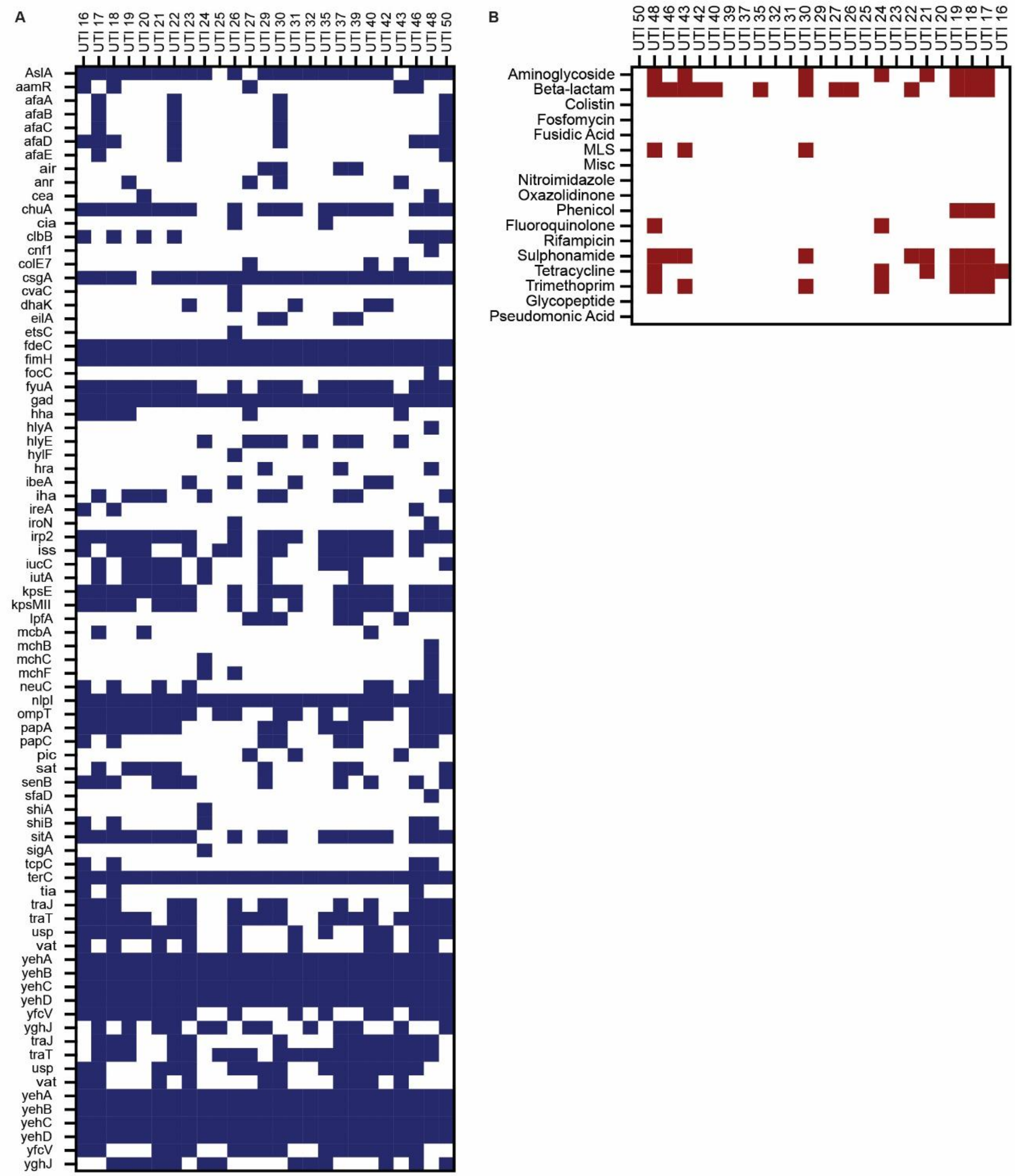

Supplemental Figure 1. *Extended virulence factors and antibiotic resistance markers for primary patient UPEC isolates.* (A) Virulence factors were determined for each strain (VirulenceFinder 2.0.5), with blue squares indicating presence of a given gene. (B) . Antibiotic resistance was also identified computationally (ResFinder 4.7.2), with maroon squares indicating resistance for a given antibiotic. (MLS = Macrolides, lincosamides, streptogramins).

bioRxiv preprint doi: https://doi.org/10.64898/2026.05.11.724322; this version posted May 12, 2026. The copyright holder for this preprint (which was not certified by peer review) is the author/funder, who has granted bioRxiv a license to display the preprint in perpetuity. It is made available under a CC-BY 4.0 International license.

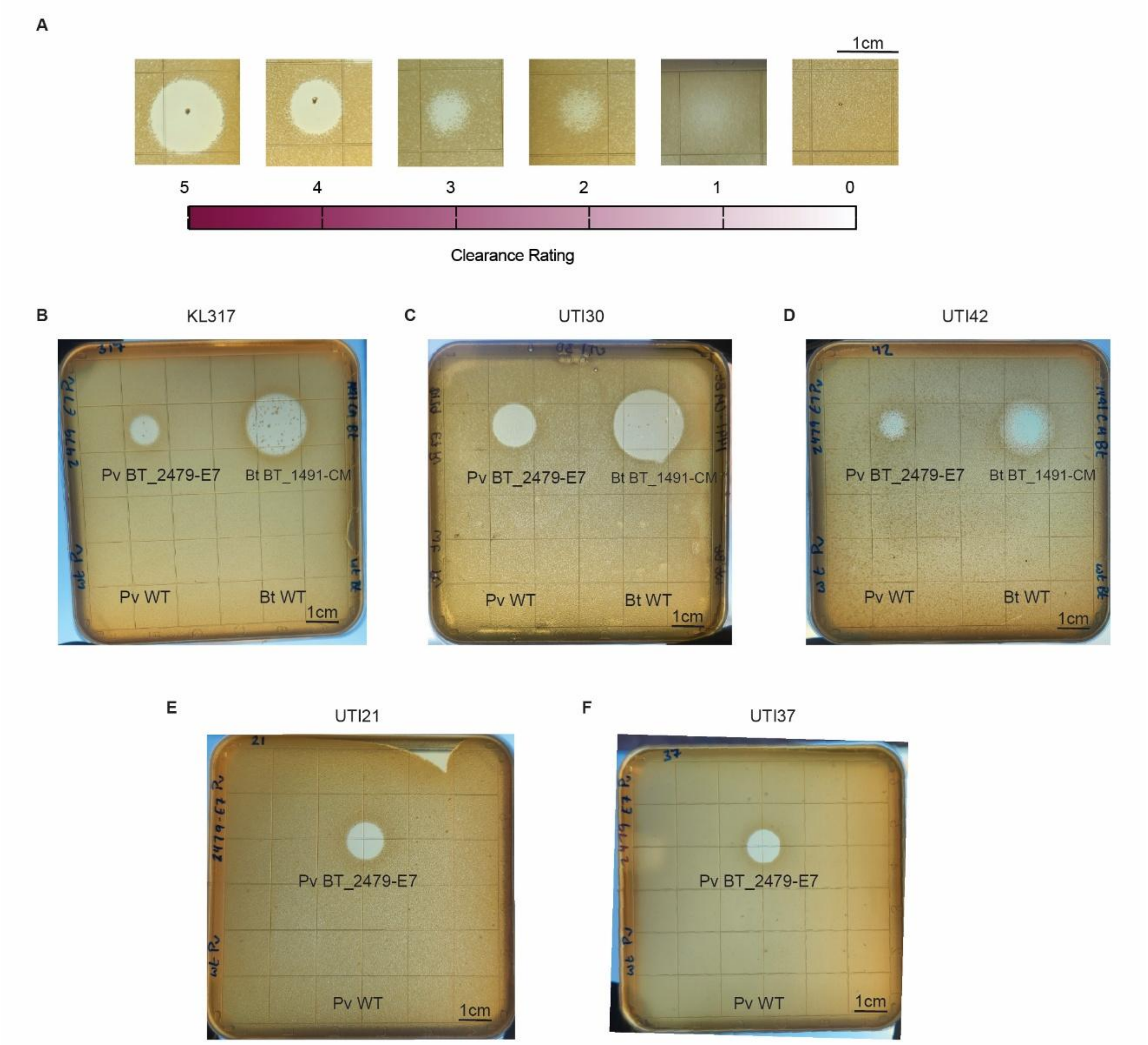

Supplemental Figure 2. *Soft agar overlay assays for determining UPEC susceptibility to colicins secreted by Bacteroidaceae.* (A) Representative images of clearance ratings for each spot as follows, with darker shading corresponding to increased clearance: no visible clearance (0), lightened area (1), hazy area (2), clearance smaller than original Bacteroidaceae spot (3), clearance equal to original spot (4), and clearance larger than original spot (5). (B-F) Representative plates for clearance of *E. coli* strains utilized in kill curve assays. For (B) KL317, (C) UTI30, and (D) UTI42, spots were placed as follows: Pv BT_2479-E7 (top left), Bt BT_1491-CM (top right), wildtype *P. vulgatus* (bottom left), and wildtype *B. thetaiotaomicron* (bottom right). For (E) UTI 21 and (F) UTI37, spots were placed as follows: Pv BT_2479-E7 (top) and wildtype *P. vulgatus* (bottom).
